# Erythrodermic mycosis fungoides with hypereosinophilic syndrome: a rare presentation

**DOI:** 10.3332/ecancer.2013.337

**Published:** 2013-08-05

**Authors:** Savita Chaudhary, Cherry Bansal, Upasna Ranga, KK Singh

**Affiliations:** 1 Department of Dermatology, Era’s Lucknow Medical College and Hospital, Lucknow 226003, Uttar Pradesh, India; 2 Department of Pathology, Era’s Lucknow Medical College and Hospital, Lucknow 226003, Uttar Pradesh, India; 3 Department of Radiodiagnosis and Imaging, Saveetha Medical College and Hospital, Thandalam, Kancheepuram, Chennai 602105, Tamil Nadu, India

**Keywords:** mycosis fungoides, hypereosinophilic syndrome, cutaneous T-cell lymphomas

## Abstract

Mycosis fungoides (MF) is the most common variant of primary cutaneous T-cell lymphoma (CTCL). It is generally associated with an indolent clinical course and characterised by well-defined clinicopathological features. Although rare, CTCLs constitute 65% of all cutaneous lymphoid malignancies, of which 50% are patients with MF. The erythrodermic variants of MF, a malignancy of mature, skin homing and clonal T lymphocytes, usually present in mid to late adulthood. Association with hypereosinophilia is important in prognosis. We report a case of erythrodermic MF with hypereosinophilic syndrome in a 22-year-old female presenting with gradually progressive intractable erythroderma with intensely pruritic multiple papules, plaques, and nodules involving more than 90% of body surface area. Diagnosis was confirmed by histopathological examination and immunophenotyping from multiple skin biopsies.

## Introduction

Mycosis fungoides (MF) is the most common type of cutaneous T-cell lymphoma (CTCL) [1, 2]. The skin is the second most common site of extranodal lymphomas after the gastrointestinal tract. MF, a prototype of CTCLs, is a peripheral epidermotropic non-Hodgkin lymphoma of low-grade malignancy that is initially present in the skin and shows clinical progression through the patch, plaque, tumour and erythrodermic stages, as well as poor survival in these progressive stages. Various advances have been made in cellular and immunologic studies by the International Society for Cutaneous Lymphomas/European Organisation of Research and Treatment of Cancer (ISCL/EORTC) to reclassify the disease accordingly since 1979 [3]. MF has many clinical variants with varied and atypical presentations, and thus has earned the title of second great mimicker after syphilis. Clinical suspicion of MF allows for early diagnosis and treatment with better patient survival.

## Case Report

**Figure 1.**
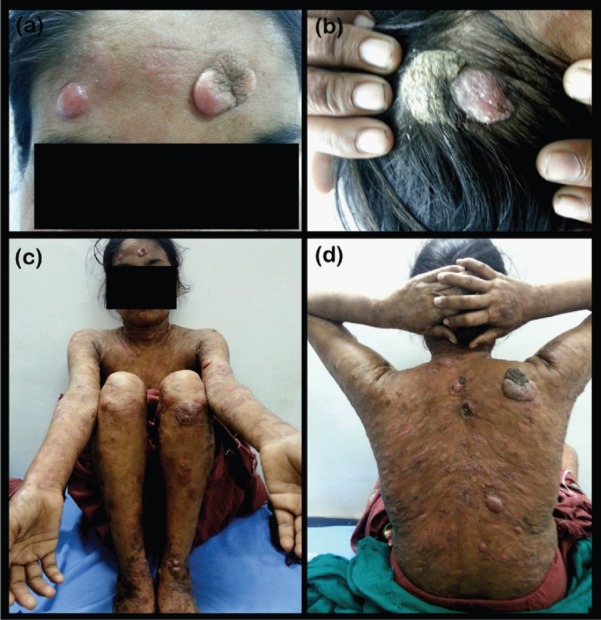
(a) The forehead of the patient, showing papulonodular and plaque lesions. (b) The scalp of the patient, showing scaly plaques and papulonodular lesions. (c) The front view of the patient, showing multiple papulonodular lesions and plaques. (d) The back view of the patient, showing papulonodular lesions, plaques, and some ulceration.

**Figure 2.**
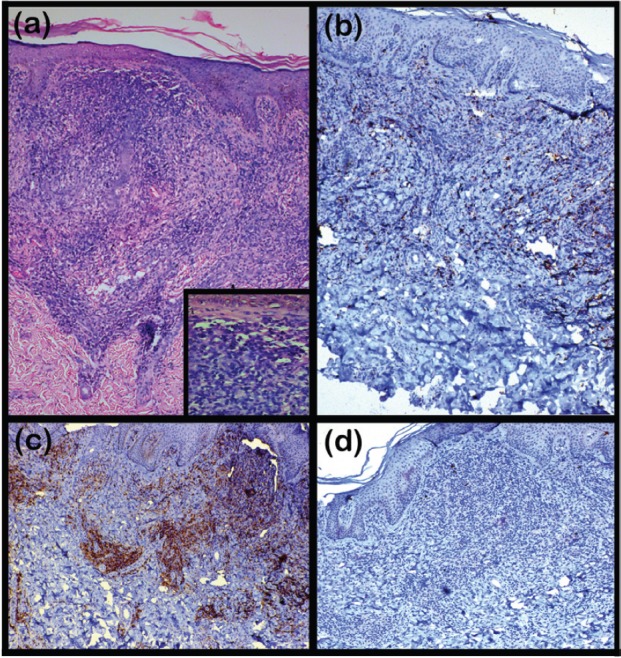
(a) A photomicrograph showing a dense diffuse lymphocytic infiltrate in the upper and mid dermis with no particular adnexotropism. The inset reveals these atypical lymphocytes mainly arranged in an interstitial pattern (H & E: 100× and 400×). (b) A photomicrograph showing lymphocytic infiltrate weakly positive for T-cell marker CD8 (IHC: 400×). (c) A photomicrograph showing lymphocytic infiltrate strongly positive for T-cell marker CD4 (IHC: 400×). (d) A photomicrograph showing negative expression of B-cell marker CD20 (IHC: 400×).

A 22-year-old female presented in the Dermatology Outpatient Department of Era’s Lucknow Medical College and Hospital, complaining of multiple itchy lesions all over the body, which were initially flat but had gradually increased in size and number over the past six months. A few of the lesions had been eroded with a foul-smelling discharge. The patient had received treatment from a local medical practitioner in the form of oral antibiotics and anti-inflammatory drugs according to medicine strips she showed us, but no detailed records were available, and she did not have any relief from this treatment. On dermatological examination, multiple plaques and nodules were distributed all over the body, including the scalp, face, chest, abdomen, and bilateral upper and lower limbs, of size ranging from 1 × 2 to 6 × 7 cm. A few lesions had well-defined margins, while others had ill-defined margins. The colour of the lesions varied from normal skin colour to erythematous [Figure 1(a)–(d)]. Excoriation marks were present all over the body. A clinical diagnosis of MF was made, and multiple skin biopsies were taken from different sites. On histopathological examination, the scanner view revealed a dense diffuse lymphocytic infiltrate in the upper and mid dermis with no particular adnexotropism. Neither was there on any areas of pale blue-staining mucin pools. Higher magnification revealed that these atypical lymphocytes were mainly arranged in an interstitial pattern. On immunohistochemistry (IHC), lymphocytic infiltrate was strongly positive for T-cell marker CD4 but not for CD8. B-cell marker CD20 did not show positive expression in epidermotropic cells [Figure 2(a)–(d)].

**Figure 3.**
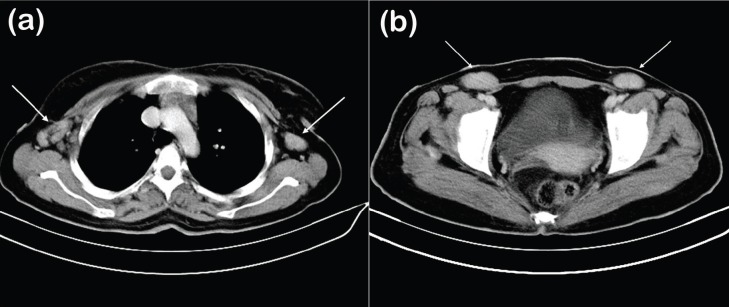
Contrast Enhanced Axial Computed Tomographic Images showed (a) bilateral enlarged axillary lymph node (white arrows) (b) Bilateral enlarged inguinal lymph nodes (white arrows).

Contrast Enhanced Axial Computed Tomographic (CECT) images showed bilateral axillary and inguinal lymph nodes enlarged (white arrows). The dimensions of axillary lymph nodes were; Left : 25 × 17.1 mm, Right: 40.6 × 16 mm and of inguinal lymph nodes were Left: 33.7 ×15 mm, Right: 40.6 × 18.1 mm [Figure 3(a) and (b)].

A haematological examination revealed the following values: haemoglobin: 10.9 g/dl (reference range 14.0–17.5 g/dl); total leucocyte count: 13,500 cells/μl ( reference range 4,500–11,000 cells/μl); neutrophils: 65% (reference range 40–75%); lymphocytes: 18% (reference range 20–45%); eosinophils: 16% (reference range 01–06%); monocytes: 01% (reference range 01–08%); and absolute eosinophil count (AEC): 2,150/μl (reference range 40–400/μl). Bone marrow aspiration was not conclusive. Liver and kidney function tests were within normal limits. No organomegaly was evident. CT-guided aspiration cytology from enlarged axillary and inguinal lymph nodes showed reactive lymphoid hyperplasia. Peripheral blood smears did not reveal any Sézary cells. According to updated ISCL/EORTC staging classification, she was diagnosed with the erythrodermic variant of MF, stage IIIA (T4N1M0B0): T4, erythroderma; N1, clinically abnormal peripheral lymph node; M0, no visceral organ involvement; and B0, absence of significant blood involvement. Based on the history, examination, histopathology and IHC, diagnosis of erythrodermic MF was made [3]. Narrow-band ultraviolet B (UVB) therapy and low-dose methotrexate resulted in improvement of the lesions and a decrease in AEC; regular follow-up visits were conducted for eight months to monitor progression to Sézary syndrome.

## Discussion

Alibert first described the classic plaque form of MF in 1806 [4]. He termed it MF because of the resemblance of the lesions to ‘mushrooms’. MF, a rare CTCL of mature skin homing clonal malignant T lymphocytes, has an annual incidence of 0.36/100,000 person–years in the United States [5]. It usually presents in mid to late adulthood (in the fifth–sixth decades of life) with a male to female ratio of 2:1. Blacks have twice the incidence compared with caucasians, as suggested by some studies. Most of the cases are diagnosed in the fifth and sixth decades of the person’s life (55–60 years of age) [4, 6, 7]. The erythrodermic variant was originally described in 1892. Erythroderma occurs as a progression from the plaque or patch stage of MF (secondary SS), or it may arise *de novo*. It differs from Sézary syndrome by the lack of an elevated number of circulating Sézary cells and often is termed pre-Sézary erythroderma*, *indicating that some of the cases eventually progress to Sézary syndrome [8].

Our patient had eosinophilia in peripheral blood, and the AEC was 2,150/μl. Since 1975, the following criteria suggested by Chusid *et al *[9] have been used for defining hypereosinophilic syndrome (HES)—(a) blood eosinophilia ≥ 1,500/mm^3 ^for longer than six months (or death before six months associated with signs and symptoms of hypereosinophilic disease), (b) lack of evidence for parasitic, allergic, or other known causes of eosinophilia and (c) presumptive signs of organ involvement, such as heart failure, gastrointestinal dysfunction, central nervous system abnormalities, fever, or weight loss that warrant special attention with regards to the potential development of disease complications. However, in 2010, Hans-Uwe Simon *et al *[10] proposed some changes in the definition to ensure optimal clinical evaluation and therapeutic decision and suggested that all patients with blood eosinophilia ≥ 1,500/mm without a discernable secondary cause (e.g., allergic diseases, drug hypersensitivity, parasitic, helminthic infestations, HIV infection and nonhaematologic malignancies) should be considered to have HES or a disorder that overlaps in definition with HES, regardless of the number and nature of affected organs or potential pathogenic mechanisms. In our case, AEC was 2,150/μl, without any signs of eosinophil-mediated organ damage or dysfunction at the time of presentation. There were no signs or symptoms of asthma or allergic rhinitis, which can lead to eosinophilia. Hence, we labelled our case ‘erythrodermic MF with HES’. Systemic methotrexate and narrow band UVB treatment resulted in improvement of the lesions, and AEC was decreased to 320/μl.

## Conclusion

Our patient was presented at an early age with erythroderma and hypereosinophilia without systemic involvement. It is important to differentiate MF from a number of other diseases such as Sézary syndrome, adult T-cell leukaemia, psoriasis, drug reactions, atopic dermatitis and other forms of erythrodermas [11]. A detailed history, thorough clinical examination and relevant investigations including IHC will improve the chances of correct and early diagnosis. Early diagnosis and treatment improve the outcome. Regular follow-up visits are required to monitor the progression of Sézary syndrome.
